# Drag reduction using bionic groove surface for underwater vehicles

**DOI:** 10.3389/fbioe.2023.1223691

**Published:** 2023-08-25

**Authors:** Shihao Zheng, Xi Liang, Jiayong Li, Yanyan Liu, Jun Tang

**Affiliations:** ^1^ School of Civil Engineering, Tianjin University, Tianjin, China; ^2^ Library of the People’s Public Security University of China, Beijing, China

**Keywords:** killer whale, groove surface, numerical simulation, drag reduction, biological modelling

## Abstract

**Introduction:** The reduction of drag is a crucial concern within the shipping industry as it directly influences energy consumption. This study addresses this issue by proposing a novel approach inspired by the unique ridge structure found on killer whale skin. The objective is to develop a non-smooth surface drag reduction method that can effectively decrease drag and improve energy efficiency for ships.

**Methods:** The study introduces a technique involving the creation of transverse bionic groove surfaces modeled after the killer whale skin’s ridge structure. These grooves are aligned perpendicular to the flow direction and are intended to modify the behavior of turbulent boundary layer flows that form around the ship’s hull. Numerical simulations are employed using the Shear Stress Transport k-ω model to analyze the effects of the proposed groove surface across a wide range of flow conditions. The research investigates the impact of various parameters, such as the width-to-depth ratio (λ/A), groove depth, and inlet velocity, on the drag reduction performance of the bionic groove surface.

**Results:** The study reveals several key findings. Optimal shape parameters for the bionic groove surface are determined, enabling the most effective drag reduction. The numerical simulations demonstrate that the proposed groove surface yields notable drag reduction benefits within the velocity range of 2∼12 m/s. Specifically, the friction drag reduction ratio is measured at 26.91%, and the total drag reduction ratio at 9.63%. These reductions signify a substantial decrease in the forces opposing the ship’s movement through water, leading to enhanced energy efficiency.

**Discussion:** Comparative analysis is conducted between the performance of the bionic groove surface and that of a smooth surface. This investigation involves the examination of velocity gradient, streamwise mean velocity, and turbulent intensity. The results indicate that the bionic groove structure effectively mitigates viscous stress and Reynolds stress, which in turn reduces friction drag. This reduction in drag is attributed to the alteration in flow behavior induced by the non-smooth surface.

**Conclusion:** The study proposes a novel approach for drag reduction in the shipping industry by emulating the ridge structure of killer whale skin. The transverse bionic groove surface, aligned perpendicular to flow direction, demonstrates promising drag reduction outcomes across diverse flow conditions. Through systematic numerical simulations and analysis of key parameters, the research provides insights into the drag reduction mechanism and identifies optimal design parameters for the groove surface. The potential for significant energy savings and improved fuel efficiency in maritime transportation underscores the practical significance of this research.

## 1 Introduction

Reducing energy consumption has always been an important objective in engineering. Due to the drag force, ships that undertake maritime traffic and transport functions consume a large amount of fuel. For underwater vehicles such as submarines, reducing the total drag by 10% can increase their cruising speed and range by approximately 3.75% under the same conditions (Ke et al., 2009). Friction drag accounts for a significant proportion of the total drag of a vehicle, and for underwater vehicles, it can be as high as 70%. New turbulent friction drag reduction techniques are significant for saving energy and obtaining high-performance ships (Li et al., 2015; Miyazaki et al., 2018; Song et al., 2018).

Inspired by the unique non-smooth surface structures of organisms in nature, bionic non-smooth surface drag reduction methods, such as the pit structures of dung beetle skin and the denticle structures of shark skin, are a new feasible means of applying bionic theory to reduce the drag force of underwater vehicles. This method involves changing the surface structure of an object to change the flow field (Choi et al., 2006; Domel August et al., 2018; Liu et al., 2020; Yu et al., 2020; Tian et al., 2021). By interfering with the development of turbulent structures within the boundary layer and reducing the loss of turbulent energy, bionic non-smooth surface drag reduction technology achieves the purpose of reducing the friction drag of the object (Lee and Lee, 2001; Zhang et al., 2011).

Recent research has shown that bionic non-smooth surfaces can effectively reduce surface frictional resistance. For instance, Dou et al. (2012) found that bionic surfaces mimicking fish scales through coating technology have a significant effect on skin friction reduction. Sasamori et al. (2014) experimentally evaluated the drag reduction effect of a three-dimensional sinusoidal riblet surface in a fully developed turbulent channel flow and obtained a drag reduction rate of 11.7% at a bulk Reynolds number of 3400. Aoki et al. (2010) investigated the aerodynamic characteristics and flow pattern of a golf ball, and found that the critical region of the golf ball shifts toward a lower Reynolds number with a smaller drag coefficient compared to that of a smooth ball. Han et al. (2008) applied a thermal embossing method to micro replicate the external morphology of shark skin and found that the maximum drag reduction efficiency reached 8.25% in experimental conditions. El-Samni et al. (2005), El-Samni et al. (2007) studied the relationship between the geometric parameters of the grooves and the drag reduction rate, and the maximum drag reduction rate reached 11% for the groove structure.

Besides, Hwang et al. (2017) proposed the use of superhydrophobic coatings to reduce the adhesion between water molecules and the wall surface, improve the hydrodynamic performance and prevent biological adhesion, thus reducing the resistance. It has self-cleaning, durability and wide application. Zhu et al. (2021) proposed a wettable bionic surface with special microstructure to achieve the purpose of drag reduction, and has the characteristics of wide applicability, self-adaptability and flexibility, biological anti-fouling and anti-biological adhesion, and environmental friendliness.

However, the disadvantages of microstructure such as difficulty in fabrication, poor sustainability, and expensive maintenance cannot be ignored. Furthermore, surface fouling caused by the adhesion of marine organisms makes it easy for the microstructure on the surface of an underwater vehicle to lose its drag reduction effect (Abarzua and Jakubowski, 1995). And the superhydrophobic coating is easily damaged by external influences, and the preparation is difficult and costly. Therefore, larger scale non-smooth structures are more suitable for underwater vehicles in extremely complex environments.

Odontocetes such as dolphins and *Orcinus orca* have high swimming speeds, which have been attributed to the drag-reducing properties of their unique skin structure (Fish, 2006; Fish et al., 2014; Hassanalian et al., 2019). Shoemaker and Ridgway (1991) studied the skin structure of seven dolphins/whale species and found that their skin was not smooth and had regular ridges in some areas. The shape parameters of ridges varied between regions and species, with wavelengths between adjacent peaks of ridges ranging from 0.4 to 2.4 mm, and amplitudes (trough to peak) ranging from 10 to 120 μm. Lang et al. (2017) designed sinusoidal transverse grooves modeled after the ridges of dolphins, and it was measured that sinusoidal grooves can delay the separation of the turbulent boundary layer.

In this particular investigation, the researchers have developed two bionic grooves inspired by the skin ridge of *Orcinus orca*. These grooves have larger dimensions compared to the natural ridges. To study the impact of different parameters of these grooves, numerical simulations were performed, and the resulting friction drag reduction was analyzed in terms of turbulence statistics. The findings of this study can be useful for the practical application of transverse grooves in reducing the drag force of underwater vehicles.

## 2 Methods

### 2.1 Design of the new bionic grooves

**FIGURE 1:**
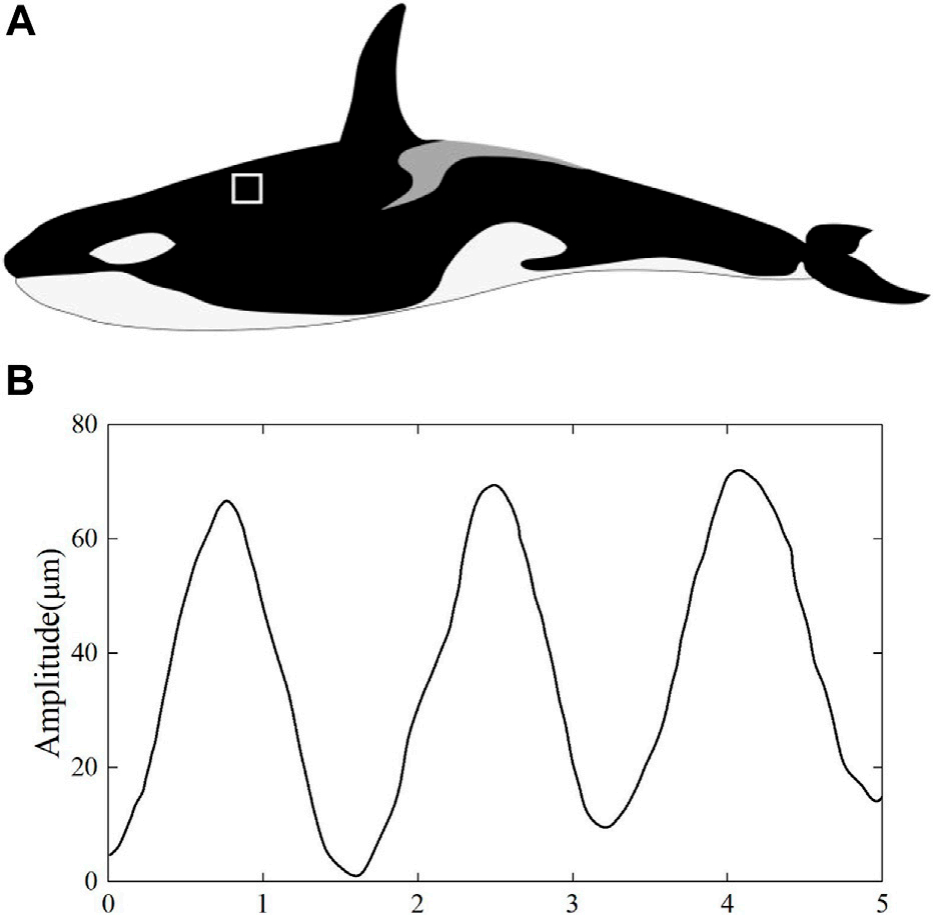
**(A)** Killer whale; **(B)** the cross-section curve of ridges.

Studying the natural surface of *Orcinus orca* replicated by silicone molds and subsequent resin casts (Wainwright et al., 2019), it was found that the ridges on the skin of *Orcinus orca* have regular shapes and distinct geometric features, which are distributed nearly perpendicular to the flow direction. The cross-sectional shape of the ridges was similar to that of a sinusoidal groove, with smooth transition between peak and trough. In Figure 1, the amplitude of the ridge is less than 80 μm and the width-to-depth ratio is greater than that of the conventional groove structure, which is about 20–30.

**FIGURE 2:**
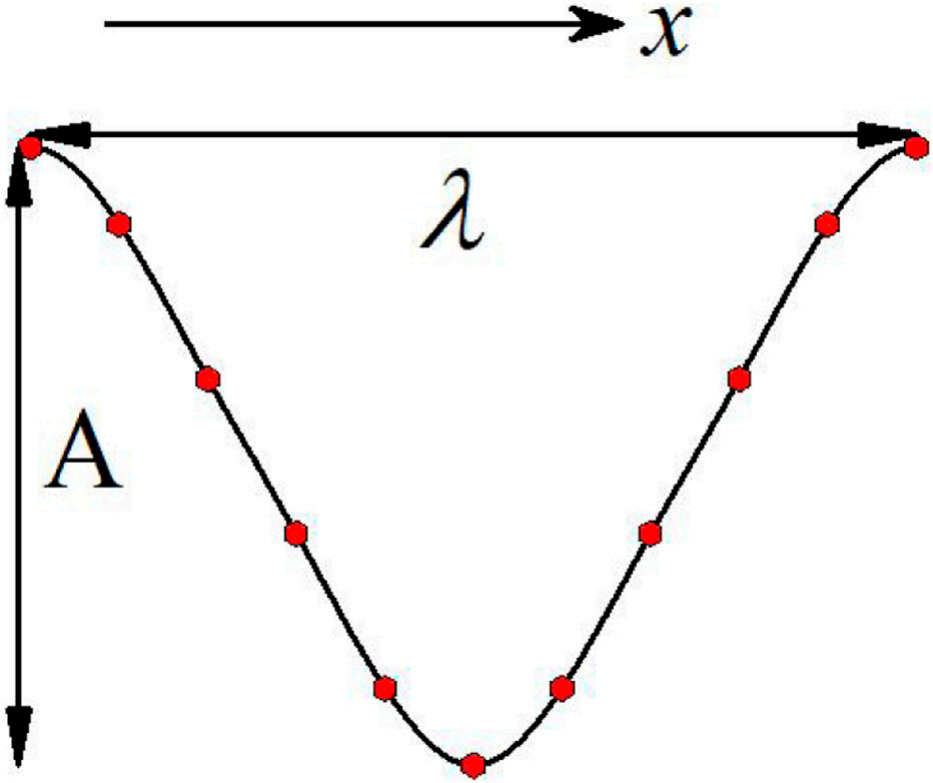
Bionic groove curve.

In this study, the skin ridge of killer whale was used as the base structure, and 11 interpolation points with the same lateral spacing were determined according to its shape characteristics, and then the numerical computation software (MATLAB) was used to generate the bionic groove curve by the cubic spline interpolation method. The interpolation points were chosen to ensure that the shape of the bionic groove approximates the ridge of killer whale. The bionic groove curve is shown in Figure 2, where A denotes the amplitude of the bionic groove, i.e., the distance between adjacent peaks and troughs, *λ* denotes the wavelength of the bionic groove, i.e., the spacing between adjacent peaks, and *x* direction is the flow direction. Compared with traditional groove structures such as rectangular and v-shaped, the new groove curve has an overall smooth transition and can reduce the pressure drag caused by the arrangement of grooves perpendicular to the flow direction to a greater extent.

**FIGURE 3:**
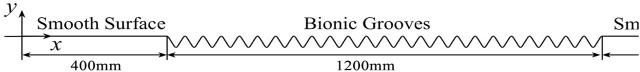
Arrangement of bionic grooves.

Novel bionic grooves were arranged on a plate to investigate their ability and mechanism to reduce friction drag. The arrangement of the bionic grooves is shown in Figure 3. The total length of the plate along the flow direction is *L* (*L = 2m*), and the transverse bionic grooves were arranged along the perpendicular to the flow direction (*x*-direction) with coordinates *y = 0* and *y = −A* corresponding to the peaks and troughs of the bionic grooves, respectively. The total length of the bionic groove area is 1.2 m, and the length of the front and rear smooth surfaces is the same.

The influence of non-smooth structure on the boundary layer fluid is an important way to reduce frictional resistance, so the amplitude of the bionic groove (*A*) should be less than the thickness of the boundary layer (*δ*) (Song et al., 2017). The thickness of the turbulent boundary layer of the smooth surface is calculated by the following equation:

$$R_{e,x}=\frac{\rho ux}{\mu }$$ (1)

$$\delta_{x}=\frac{0.37x}{{R_{e,x}}^{1/5}}$$ (2)

where *R*_*e,x*_ is the Reynolds number at the calculation point and *δ*_*x*_ is the thickness of the boundary layer at the calculation point; *ρ* is the density of the fluid; *u* is the flow velocity of the fluid; and *μ* is the dynamic viscosity of the fluid. In this study, the fluid medium is liquid water, and the density of liquid water (*ρ*) is 998.2 kg/m^3^, dynamic viscosity (*μ*) is 0.001003, and the flow velocity range is 2–12 m/s. The Reynolds number range in the bionic groove region is 7.96 × 10^5^ to 1.91 × 10^7^, ensuring that its flow state is turbulent. The thickness of the smooth surface boundary layer at the corresponding location in the bionic groove region is shown in Table 1. According to Table 1, the bionic groove should meet the amplitude *A* ≤ 6.8 mm.

**TABLE 1:** Turbulent boundary layer thickness of the smooth surface.

*u* (m/s)	*x* (mm)	*δ*_*x*_ (mm)
2	400	9.8
	1600	29.6
4	400	8.5
	1600	25.8
8	400	7.4
	1600	22.5
12	400	6.8
	1600	20.7

Although the ridges of *Orcinus orca* satisfy the scaling requirements for non-smooth surface drag reduction, they have the same microstructural drawbacks. Therefore, the ridges of *Orcinus orca* were appropriately scaled up according to the bionic principle to ensure the drag reduction performance of the new bionic grooves while making them possible for practical application. To study the effect of the variation of the geometric parameters of the bionic grooves on the drag reduction, 9 types of grooves were designed with the geometric parameters shown in Table 2.

**TABLE 2:** Geometric parameters of bionic grooves.

Groove type	A (mm)	Λ (mm)	λ/A
1	4	80	20
2	4	100	25
3	4	120	30
4	2	40	20
5	2	50	25
6	2	60	30
7	1	20	20
8	1	25	25
9	1	30	30

### 2.2 Numerical simulation methods

In the present study, the numerical simulations are carried out using the SST (shear stress transport) turbulence model, with the SIMPLE (Semi Implicit Method for Pressure Linked Equations) algorithm. The discrete format used for the numerical simulation is the second-order upwind scheme. And the physical parameters of incompressible liquid water are kept constant during the calculation. The *SST k-ω* model is a deformation of the standard *k*-*ω* model, which incorporates cross-diffusion originating from the *ω* equation, and the turbulent viscosity takes into account the propagation of turbulent shear stresses. Moreover, it has higher accuracy for simulating near-wall flow, inverse pressure gradient flow, and turning. The equations of the *SST k-ω* model are as follows:

$$\frac{\partial }{\partial t}\left(\rho k\right)+\frac{\partial }{\partial x_{i}}\left(\rho U_{i}k\right)=\frac{\partial }{\partial x_{i}}\left[\left(\mu +\frac{\mu_{t}}{\sigma_{k}}\right)\frac{\partial k}{\partial x_{j}}\right]+G_{k}+S_{k}-Y_{k}$$ (3)

$$\frac{\partial }{\partial t}\left(\rho \omega \right)+\frac{\partial }{\partial x_{i}}\left(\rho U_{i}\omega \right)=\frac{\partial }{\partial x_{i}}\left[\left(\mu +\frac{\mu_{t}}{\sigma_{\omega }}\right)\frac{\partial \omega }{\partial x_{j}}\right]+G_{\omega }+D_{\omega }+S_{\omega }-Y_{\omega }$$ (4)

where *k* is the turbulent kinetic energy, *ω* is the specific dissipation rate, *μ*_*t*_ is the turbulent kinematic viscosity coefficient, *G*_*k*_ and *G*_*ω*_ are the turbulent kinetic energy generated by the mean velocity gradient and *ω* equations, respectively, *σ*_*k*_ and *σ*_*ω*_ represent the turbulent Planter constants for *k* and *ω*, respectively, *S*_*k*_ and *S*_*ω*_ are the correlation source terms, *Y*_*k*_ and *Y*_*ω*_ represent the divergence terms for *k* and *ω*, respectively, and *D*_*ω*_ are the turbulent cross terms.

The expressions of the turbulent eddy viscosity coefficient for the *SST k-ω* turbulence model are as follows:

$$\mu_{t}=\frac{a_{1}k}{\max\left(a_{1}\omega ,\Omega F_{2}\right)}$$ (5)

In the above equation, *a*_1_ is Bradshaw’s constant with a value of 0.31; Ω is the absolute value of the vortex; *F*_2_ is a switching function, and in the *SST k-ω* turbulence model, *F*_2_ takes a value of 1 in wall turbulent and *F*_2_ takes 0 in free shear flow. The expression *F*_*2*_ is:

$$F_{2}=\tanh\left(\arg_{2}^{2}\right)$$ (6)

$$\arg_{2}=\max\left(\frac{2\sqrt{k}}{0.09\omega y},\frac{500\mu }{\rho y^{2}\omega }\right)$$ (7)

### 2.3 Boundary conditions and mesh

**FIGURE 4:**
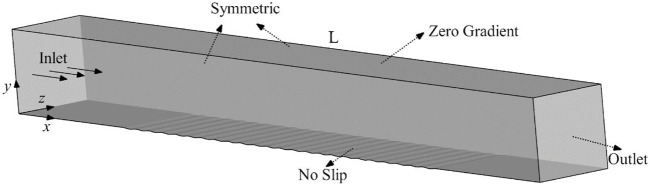
Schematic of the 3D geometry of the flow domain used in numerical simulation.

A 3D geometric model of the flow domain used in numerical simulation was established according to the bionic groove arrangement in Figure 3, and a schematic of the geometry and the coordinate system used is shown in Figure 4. The velocities in the *x*, *y*, and *z* directions are denoted by *u*, *v*, and *w*, respectively, and the total length of the flow domain along the flow direction is denoted by *L(L = 2m).* The width and height of the flow domain is always 0.2 m.

The boundary conditions of flow domain are shown schematically in Figure 4. At the inlet (at the plane *x* = 0), the flow enters the flow domain with a uniform velocity. The groove surface that can be replaced with the smooth surface is a no-slip wall at the bottom of the flow domain, the upper surface has a zero pressure and no velocity gradient across it and the outlet (at the plane *x* = *L*) has zero velocity gradient across it and zero pressure. The two side walls are symmetric boundary conditions.

**FIGURE 5:**
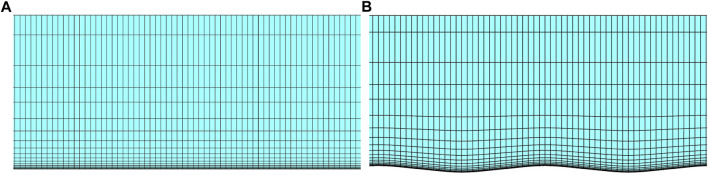
**(A)** Smooth surface near-wall grid, and **(B)** groove surface near-wall grid.

The near-wall grids of the groove surface and smooth surface are shown in Figure 5, and the flow domain grids near the walls were encrypted to improve the accuracy of the numerical simulation. The first layer of the grid was located at the viscous sublayer of the boundary layer and satisfied *y* ^*+*^ close to 1, $y^{+}=\left(\tilde{y}\sqrt{\rho \tau_{w}}\right)/\mu$ , where ỹ is the normal distance from the surface and *τ*_*w*_ is the wall shear stress. The grid of the entire flow domain was refined to verify that the computational results were independent of the number of grids. As a result, the friction drag of the smooth surface was only reduced by 0.1%, when the number of grids increased from 1 × 10^6^ to 1.2 × 10^6^. To improve the computational efficiency, the number of grids for subsequent calculations was set to 1 × 10^6^.

**FIGURE 6:**
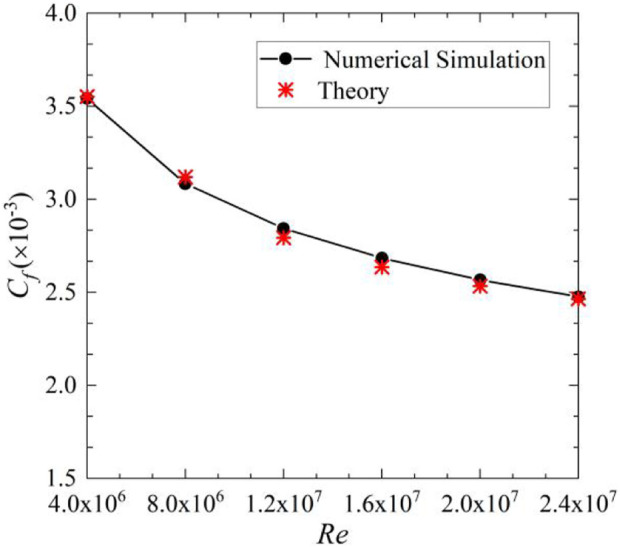
Friction drag coefficient of smooth surface.

Moreover, under the simulation setting, the simulation reliability verification was carried out. Figure 6 shows the simulated and theoretical equation ( $C_{f}=0.074/{Re}^{1/5}$ ) calculated values of the friction drag coefficient for different flow conditions on the smooth surface. The simulated values are in good agreement with the theoretical values, and the relative errors are within ±2%, indicating that the simulation model is reliable.

## 3 Results and discussion

### 3.1 Drag reduction performance analysis

In this study, the friction drag reduction ratio and total drag reduction ratio of the bionic groove surface were analyzed, and they can be calculated by the following equations:

$$R_{f}=\frac{N_{f}-N_{fn}}{N_{f}}\times 100\%$$ (8)

$$R_{t}=\frac{N_{t}-N_{tn}}{N_{t}}\times 100\%$$ (9)

where *R*_*f*_ is the friction drag reduction ratio, *R*_*t*_ is the total drag reduction ratio, *N*_*f*_ is the friction drag of the smooth surface, *N*_*fn*_ is the friction drag of the groove surface, *N*_*t*_ is the total drag of the smooth surface, *N*_*tn*_ is the total drag of the groove surface. The smooth surface is selected as a reference. *R*_*f*_ > 0 indicates that the goal of reducing friction drag is achieved, whereas *R*_*f*_ < 0 means that the friction drag is increased, *R*_*t*_ is also like that.

**FIGURE 7:**
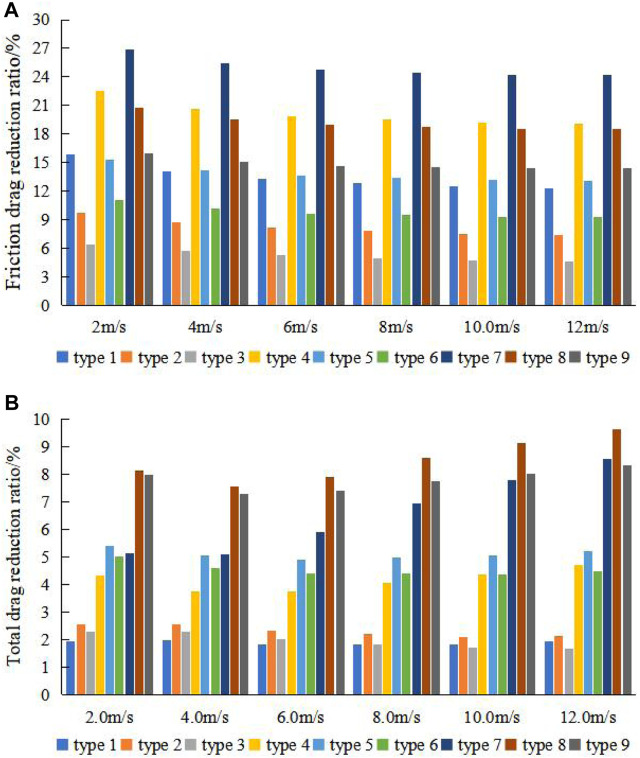
**(A)** Friction drag reduction ratio and **(B)** total drag reduction ratio of bionic groove surfaces at different velocities.

The drag reduction ratio of all bionic groove surfaces at different velocities are shown in Figure 7. Figure 7 shows that all groove surfaces achieve the goal of reducing the total drag by reducing the surface friction drag. When the width-to-depth ratio and flow velocity are the same, the smaller the depth, the better the drag reduction effect of the bionic groove surface. And when the depth and flow velocity are the same, with the increase of the width-to-depth ratio, the friction drag reduction ratio of the groove surface decreases, while its pressure drag also gradually decreases, resulting in the total drag reduction ratio the first rise and then decline, the best width to depth ratio is 25.

The trend of the friction drag reduction ratio is the same for all groove surfaces, and only numerical differences exist. The trend exhibited is that the friction drag reduction ratio gradually decreases as the flow velocity increases, but the groove surface still obtained a friction drag reduction rate of 24.16% at the velocity of 12 m/s. In addition, the peak friction drag reduction ratio is achieved at the velocity of 2 m/s for all groove surfaces.

### 3.2 Drag reduction mechanism analysis

For high Reynolds number turbulent flow fields, the wall shear stress of plate is influenced by both viscous stress and turbulent Reynolds stress. In the near-wall region, the viscous stress plays a major role, and away from the wall, the Reynolds stress becomes the dominant force due to the pulsation of a large number of fluid micro clusters. Since groove type 8 has a better friction drag reduction performance, the changes of viscous stress and turbulent Reynolds stress in the flow field are analyzed in terms of boundary layer thickness, velocity gradient and turbulent intensity to explore the drag reduction mechanism.

#### 3.2.1 Velocity and pressure contour analysis

**FIGURE 8:**
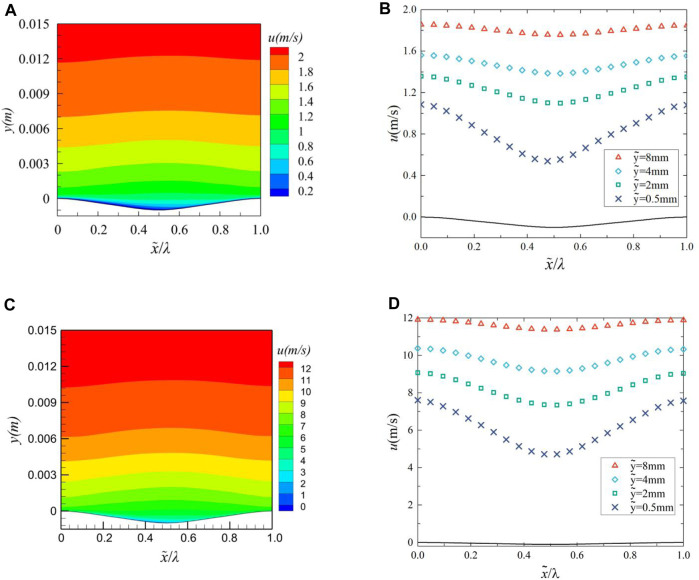
Streamwise velocity contour of the middle cross-section at **(A)** 2 m/s and **(C)** 12 m/s, variation of streamwise velocity at **(B)** 2 m/s and **(D)** 12 m/s.

Periodic bionic groove surfaces cause changes in turbulent flow. Analysis of the flow field in the middle region of groove surface 8 at Reynolds number of 3.98 × 10^6^ (*u* = 2 m/s) and 2.39 × 10^7^ (*u* = 12 m/s) reveals periodic variations in velocity and pressure in the near-wall region. Figures 8A, C shows the streamwise velocity(*u*) contour in one groove range, when x = 0.5, Streamwise velocity contour of the middle cross-section at (a) 2 m/s and (c) 12 m/s have minimum values of −0.0016 and −0.001, respectively; and Figures 8B, D shows the streamwise velocity in one groove range for four different normal (y-direction) locations, when x = 0.5, the variation of streamwise velocity at (b) 2 and (d) 12 m/s have minimum values of 4.61 and 4.58. The transverse coordinate is dimensionless using the bionic groove wavelength *λ*. From Figure 8, it can be analyzed that the streamwise velocity gradient within the boundary layer gradually decreases as the thickness of the boundary layer gradually increases from peak to trough, and thus the viscous shear stress gradually decreases. From trough to peak, the opposite trend is shown. And the trend of the streamwise velocity is nearly synchronized with the waveform undulation, and the streamwise velocity reaches a maximum value at the peak and a minimum value at the trough, and the change is more obvious the closer to the surface.

**FIGURE 9:**
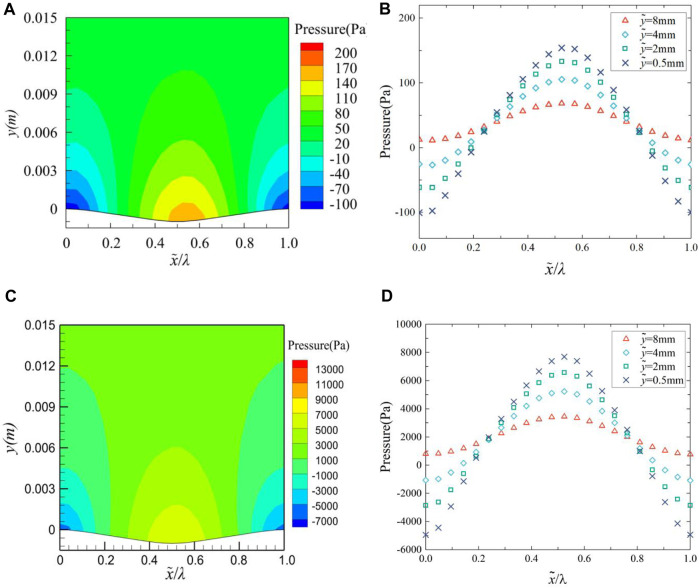
Pressure contour of the middle cross-section at **(A)** 2 m/s and **(C)** 12 m/s, variation of pressure at **(B)** 2 m/s and **(D)** 12 m/s.

The pressure contour over a groove range is shown in Figure 9A, and the pressure variation at four different normal (*y*-direction) locations over a groove range is shown in Figure 9B. In Figure 9, when x = 0.49, Pressure contour of the middle cross-section at (a) 2 m/s and (c) 12 m/s have minimum values of −0.001 and −0.0006, respectively; When x = 0.5, the variation of streamwise velocity at (b) 2 m/s and (d) 12 m/s have minimum values of 0.52 and 4.48, respectively. Analysis of Figure 9 reveals that a low-pressure area appears near the peak of the groove, or even a negative pressure near the surface, while a high-pressure area is found at the trough of the groove. The pressure variation also exhibits periodicity with a trend, that is, exactly opposite to the waveform undulation, which is consistent with the pressure variation trend measured by Zilker et al. (1977).

In summary, the change of pressure gradient induces the change of streamwise velocity, from the peak to the trough, due to the flow of the fluid against the pressure gradient, the fluid particles in the boundary layer are affected by viscosity and the retardation of the inverse pressure gradient, then the kinetic energy is rapidly lost. So that the turbulent stress decreases and the fluid accumulates in the trough, the viscous shear stress gradually decreases. From trough to peak, boundary layer fluid flows at an accelerated rate along the pressure gradient, which increases the kinetic energy of the fluid particle and makes the viscous effect between the fluids more obvious, so the turbulent stress increases, resulting in the increase of viscous shear stress.

#### 3.2.2 Velocity vector and average velocity profiles analysis

**FIGURE 10:**
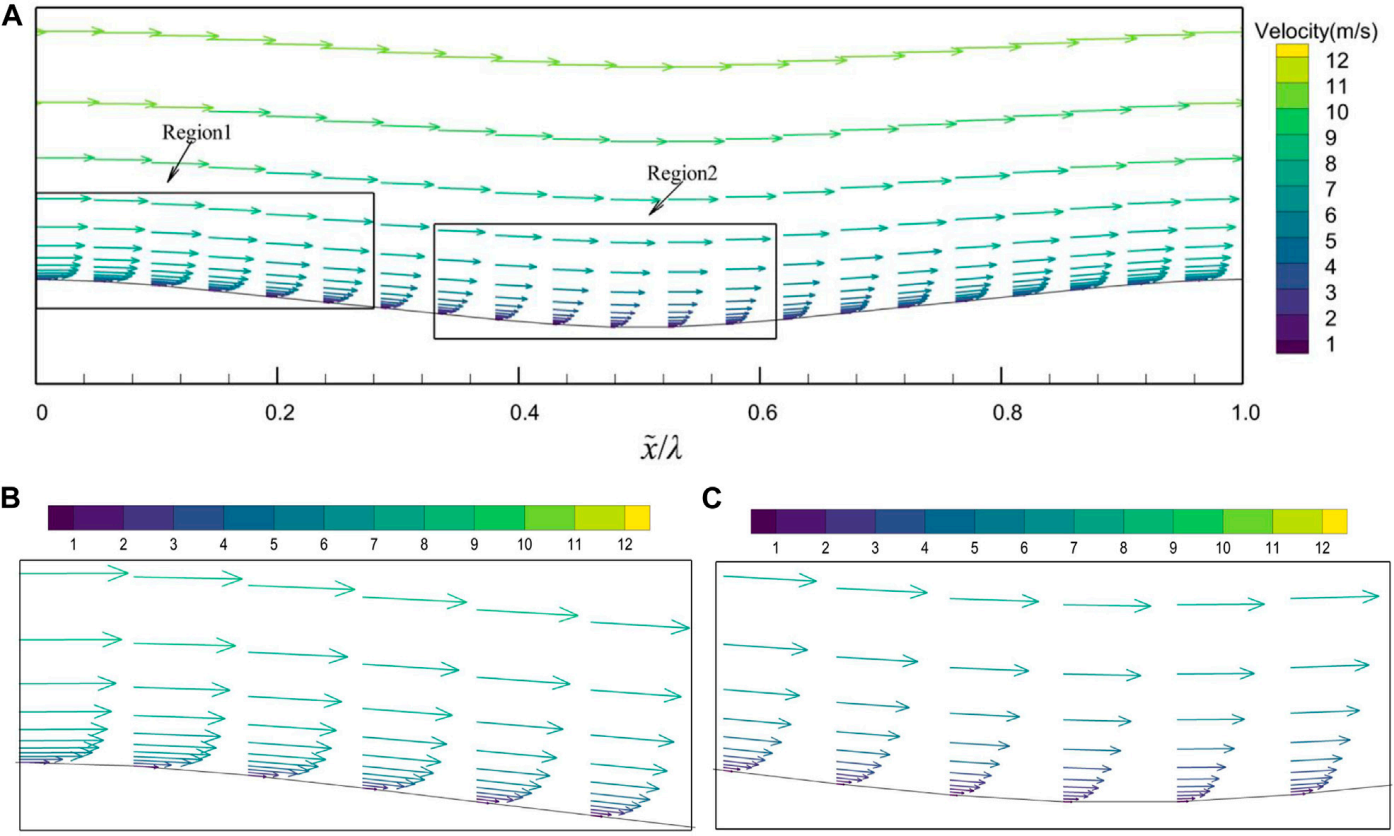
Velocity vector of the middle cross-section at *u* = 12 m/s. **(A)** a groove area, **(B)** enlargement of region 1, **(C)** enlargement of region 2.

The variation of the cross-sectional (*x*-direction) velocity vector over the range of a groove is shown in Figure 10. In Figure 10, when x = 0.55, the Velocity vector of the middle cross-section at u = 12 m/s has a minimum value of 0.85. It is found that the bionic groove surface can not only change the flow direction and produce the velocity component in y direction, but also significantly change the velocity gradient near the surface, although no backflow phenomenon is observed in the trough region. The velocity gradient on the groove surface varies periodically as follows: from the peak to the trough, the velocity gradient gradually decreases under adverse pressure gradient, which leads to a decrease in viscous stress, and from the trough to the peak, the velocity gradient gradually increases under favorable pressure gradient. The effect of this change in velocity gradient on viscous stress is one of the reasons for the reduction of friction resistance on the groove surface.

**FIGURE 11:**
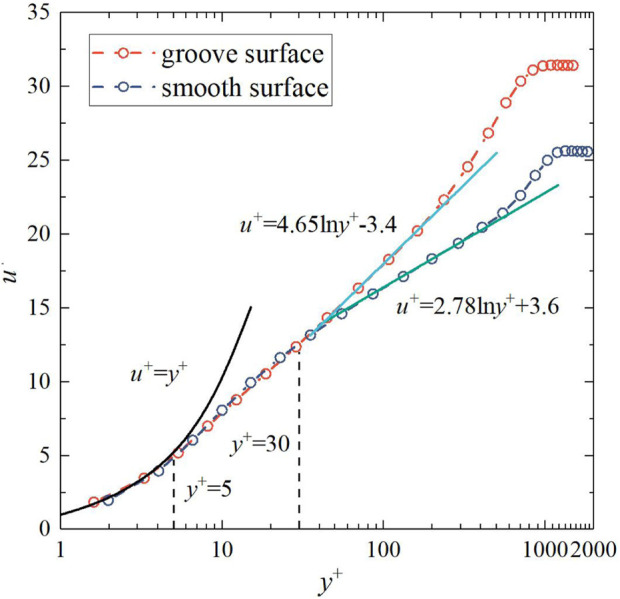
Mean velocity distribution profiles in wall-normal direction over the groove and smooth surface.

To study the streamwise velocity variation caused by a single bionic groove structure as a whole, the streamwise velocities at the same normal position in the range of adjacent peaks are averaged. The distribution of the dimensionless streamwise mean velocity ( $u^{+}=u/\sqrt{\tau_{\omega }/\rho }$ ) along the wall-normal direction for the groove and smooth surface is given in Figure 11. As with the smooth surface, the streamwise mean velocity on the groove surface has zoning characteristics along the normal direction, satisfying linear and logarithmic distributions in different regions, respectively. In the near-wall region (*y* ^*+*^ *<30*), there is almost no difference between the groove surface and the smooth surface, and in the region where *y* ^*+*^ *<5, u* ^*+*^ is linearly distributed for both surfaces, satisfying u^+^ = y^+^. And in the region where *y* ^*+*^ *>30*, the distribution of *u* ^*+*^ along the normal direction of the groove surface differs significantly from that of the smooth surface, but still satisfies the law of logarithmic distribution. Moreover, when the value of *y* ^*+*^ is the same, the *u* ^*+*^ of the groove surface is significantly higher than that of the smooth surface, and there is a significant upward shift in the log-law region and the outer region of the dimensionless streamwise mean velocity distribution curve on the groove surface.

In summary, a single bionic groove structure as a whole leads to an outward shift of the log-law region and outer region of the dimensionless streamwise mean velocity distribution curve, thus reducing Reynolds stress of the flow field, which is in line with the typical characteristics of drag-reducing surfaces (Choi et al., 1993; Wang et al., 2000).

#### 3.2.3 Turbulent intensity and Reynolds shear stress analysis

The magnitude of the turbulent velocity fluctuations can reflect the magnitude of Reynolds stress, and the turbulent intensity is the ratio of the square root of the pulsation velocity to the time-averaged velocity, so the turbulent intensity can also reflect the magnitude of pulsation velocity and Reynolds stress.

**FIGURE 12:**
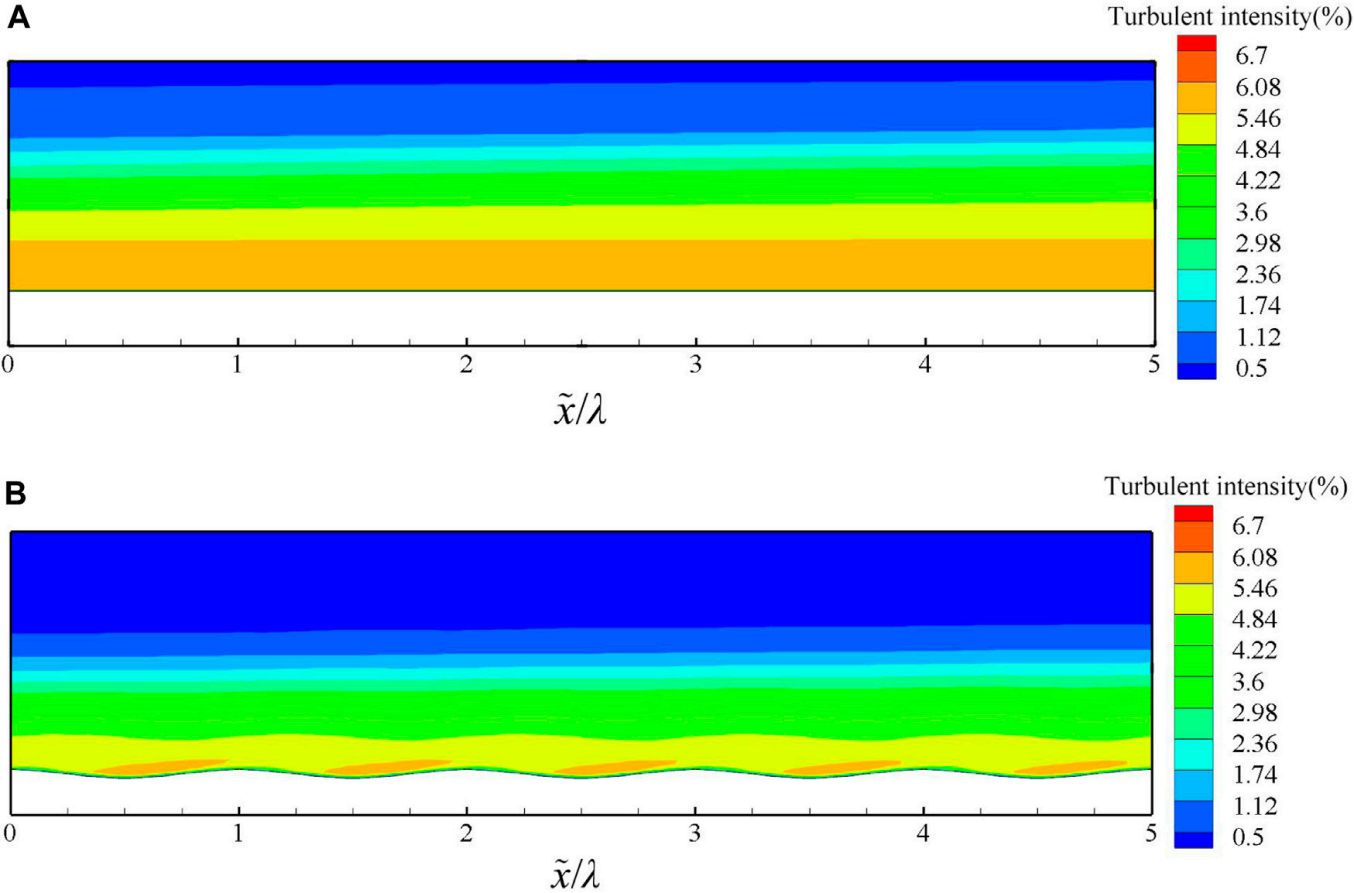
Turbulent intensity contour of **(A)** smooth surface and **(B)** groove surface.

The turbulent intensity contour of groove surface and smooth surface is shown in Figure 12. In a period of x = 0 to 1 in Figure 12B, the turbulence intensity near the wall increases to 6.05 at x = 0.37 to 0.91. Obviously, the effect of the bionic groove makes the turbulent intensity of flow field decrease rapidly away from the wall, and the turbulent intensity of the groove surface is overall lower than that of the smooth surface. While the higher turbulent intensity on the windward side inside the bionic groove may be caused by the enhanced turbulent pulsation due to the effect of the favorable pressure gradient.

**FIGURE 13:**
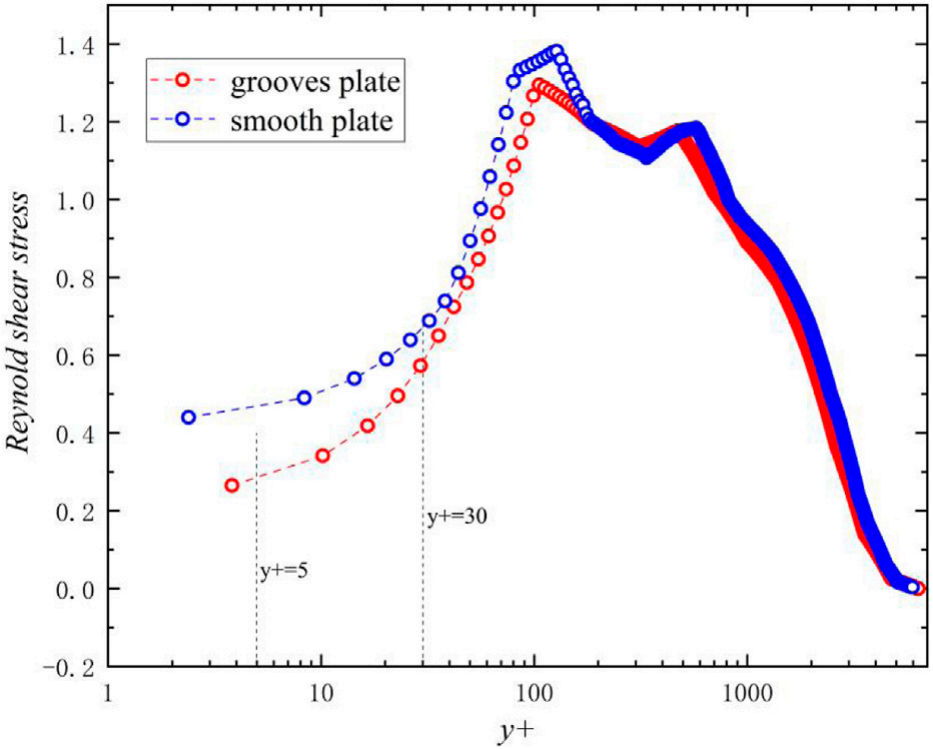
Reynolds stress curve of grooved surface and smooth surface.

The Figure 13 shows the Reynolds shear stress curve of the groove surface and the smooth surface in a single bionic groove structure, it can be seen from the figure that the overall change trend of the groove surface and the smooth surface is consistent. The Reynolds shear stress on the groove surface is always smaller than that on the smooth surface at *y* ^*+*^ *<100*. In the near-wall area (*y* ^*+*^ *<30*) the Reynolds shear stress on the groove surface is smaller than that on the smooth surface when it is closer to the wall, which indicates that the velocity pulsation of the boundary laminar flow field is suppressed by the groove surface, and the effect on the near-wall flow field is more obvious. The Reynolds shear stress of smooth surface and groove surface reaches the peak at *y* ^*+*^ *≈100,* and begins to close at *y* ^*+*^ *≈200*. When approaching the second peak, the Reynolds shear stress on the groove surface and the smooth surface gradually decreases as it moves away from the wall, indicating that the suppression effect of the groove surface on turbulent pulsation gradually decreases.

**FIGURE 14:**
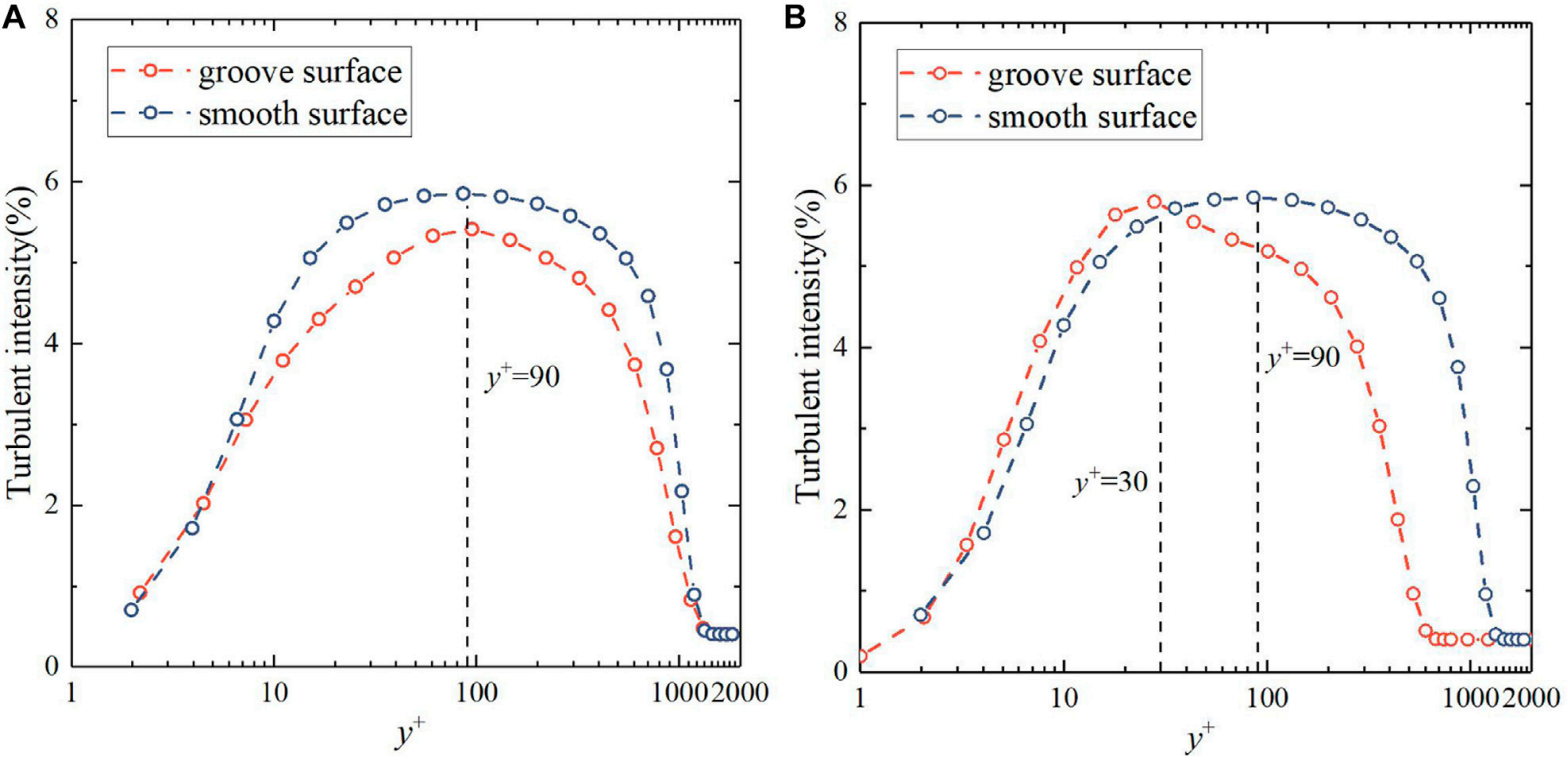
Turbulent intensity at **(A)** the peak and **(B)** trough at u = 2 m/s (Re = 3.98 × 106).

Figure 14 show the curves of turbulent intensity along the normal direction at the peak and adjacent trough of the bionic groove surface. From Figure 14A, it can be seen that the trend of turbulent intensity at the peak of the bionic surface is the same as that of the corresponding smooth surface, and the *y* ^*+*^ corresponding to the maximum turbulent intensity is similar. When *y* ^*+*^ is the same, the turbulent intensity at the peak of the groove surface is lower than that of the corresponding smooth surface, indicating that the turbulent pulsation intensity at the peak of the groove surface is lower than that of the corresponding smooth surface.

The maximum turbulent intensity at the trough of the groove surface is close to that of the smooth surface. Meanwhile, the turbulent intensity at the trough of the groove surface first reaches the peak value and then decreases rapidly, appearing quite different compared with the smooth surface, which should be caused by the presence of a high turbulent intensity region on the windward side of the bionic groove.

When far from the surface, the turbulence statistics of the flow field on the bionic groove surface and the smooth surface are not different, and the turbulent motion away from the surface is almost unaffected by the surface conditions, which is consistent with the findings of Hudson et al. (1996), Nakagawa et al. (2003). In summary, the bionic groove structure reduced the turbulence intensity near the wall area, and inhibited the velocity pulsation of the groove surface, reduced the Reynolds shear stress in the flow field near the wall, weakened the exchange of momentum and kinetic energy during fluid motion, thus achieving the effect of reducing surface friction drag.

#### 3.2.4 Wall shear stress analysis

**FIGURE 15:**
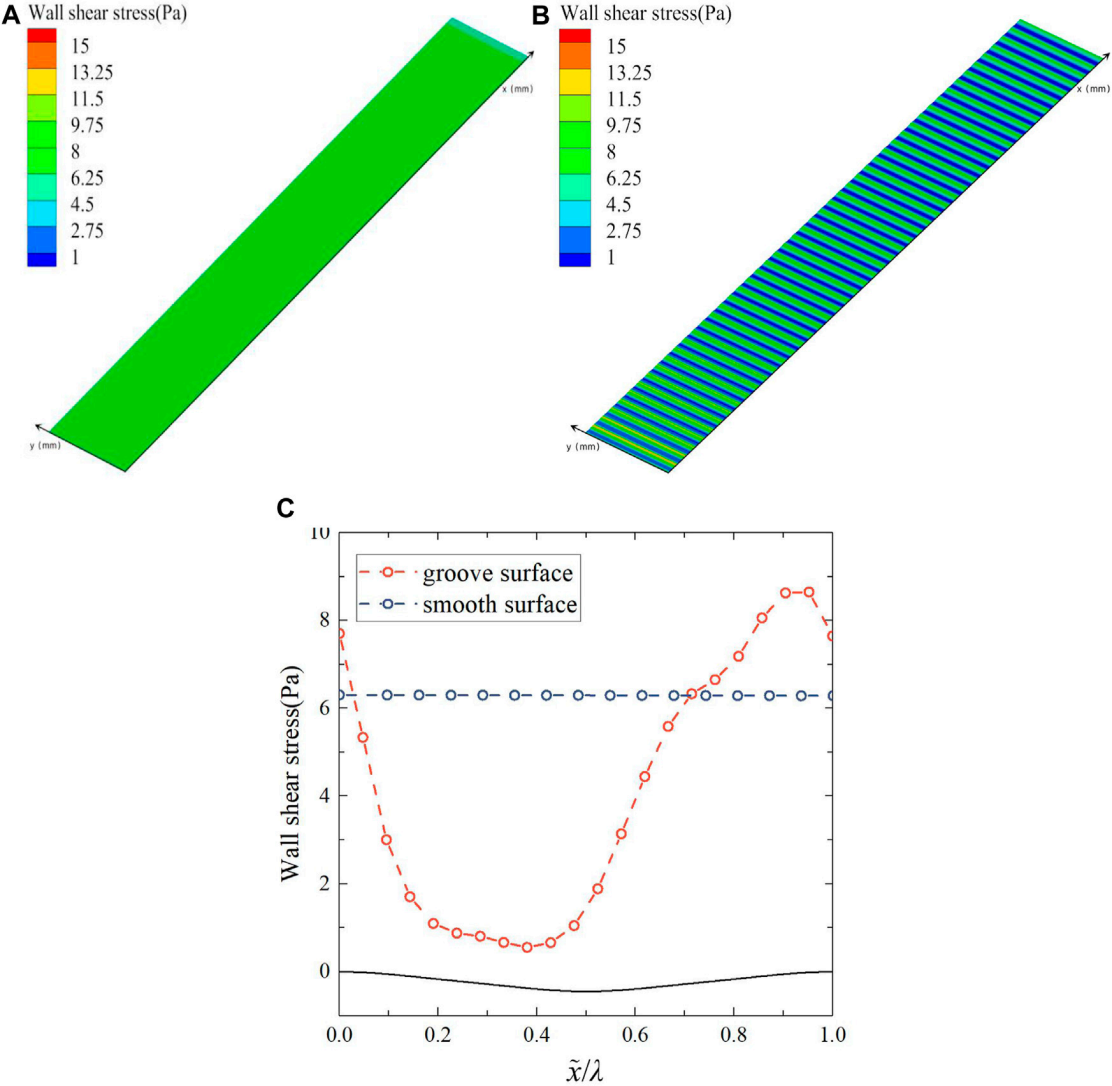
**(A)** Wall shear stress contour of smooth surface, **(B)** wall shear stress contour of groove surface, and **(C)** wall shear stress curves.

The wall shear stress contours and curves of the groove and smooth surface are given in Figure 15. In Figure 15, when x = 0.39, Wall shear stress has a minimum value of 0.46; At x = 0.91, the Wall shear stress has a maximum value of 8.6. The shear stress on the groove surface appears to change periodically, from the peak to the trough, the wall shear stress decreases and then increases, and from the trough to the peak it shows the opposite trend. The trend of the shear stress on the groove surface is not exactly synchronized with the waveform undulation of the groove, and there is a certain phase difference. Compared with the smooth surface, the shear stress in the peak region of the groove surface is higher than that of the smooth surface, and the shear stress in the trough region is lower than that of the smooth surface, which coincides with the variation of the viscous stress and Reynolds stress. The overall shear stress on the groove surface is significantly lower than that on the smooth surface, which is the direct cause of the lower friction drag on the groove surface than on the smooth surface.

To sum up, the flow field analysis on the bionic groove surface demonstrates that the streamwise velocity, pressure, and velocity gradient change periodically, such that the fluid flows under adverse pressure gradient from the peak to the trough. As a result, the streamwise velocity and velocity gradient gradually decrease, leading to a reduction in the viscous stress in the near-wall region, while the opposite trend is observed from the trough to the peak.

The study investigates the influence of individual bionic groove as a whole on the dimensionless streamwise mean velocity, and it is found that there is little variability between the groove surface and the smooth surface in the near-wall region (*y* ^*+*^ *<30*), but the groove structure leads to an upward shift in the log-law and outer regions of the mean velocity profile. This observation suggests that the fully developed turbulent flow region is relatively far from the wall. The comparison of the turbulent intensity of the bionic groove surface with that of the smooth surface reveals that the bionic groove structure reduces the turbulent intensity of the flow field and suppresses the velocity pulsation, thus reducing the Reynolds stress.

It can be concluded that compared with the smooth surface, the viscous stress and Reynolds stress of the groove surface are significantly reduced, resulting in the overall shear stress is significantly less than that of the smooth surface. Consequently, this corresponds to the drag reduction effect observed on the groove surface.

## 4 Conclusion

*Orcinus orca* possess a distinctive skin structure characterized by regular ridges that cover most of their bodies. This paper presents a design for nine new transverse bionic grooves with larger geometric scales that are inspired by the killer whale skin ridges. In comparison with the traditional groove structure, the new bionic groove has a greater width-to-depth ratio and hence larger geometric scale at the same depth. The numerical simulation results reveal that the drag reduction effect improves as the depth of the bionic groove surface decreases, with the optimal width-to-depth ratio being 25. Over the flow velocity range of 2–12 m/s, the friction drag reduction ratio decreases gradually as the flow velocity increases, but the trend tends to level off, with the optimal friction drag reduction ratio reaching 26.91% and the total drag reduction ratio reaching 9.63%.

Therefore, the transverse bionic grooves designed in this paper has a broad engineering application prospect. The next step is to explore the drag reduction effect of applying the new bionic groove to a full-size model at high Reynolds number for an aircraft or submarine model. This will provide a theoretical basis for the practical application of transverse bionic grooves.

## Data Availability

The original contributions presented in the study are included in the article/supplementary material, further inquiries can be directed to the corresponding author.
